# Investigation of IL-23 (p19, p40) and IL-23R identifies nuclear expression of IL-23 p19 as a favorable prognostic factor in colorectal cancer: a retrospective multicenter study of 675 patients

**DOI:** 10.18632/oncotarget.2069

**Published:** 2014-06-06

**Authors:** Melina Helbling, Anne Lukesch, Aladin Haimovici, Eva Karamitopoulou, Martin D. Berger, Marion Hädrich, Makhmud Mallaev, Beat Schnüriger, Viktor H. Koelzer, Heather Dawson, Markus Borner, Rupert Langer, Robert Rosenberg, Ulrich Nitsche, Daniel Inderbitzin, Alessandro Lugli, Mario P. Tschan, Inti Zlobec

**Affiliations:** ^1^ Institute of Pathology, University of Bern, Bern, Switzerland; ^2^ Department of Visceral Surgery and Medicine, Bern University Hospital, Bern, Switzerland; ^3^ Department of Medical Oncology, Bern University Hospital, Bern, Switzerland; ^4^ Department of Oncology, Hospital Centre Biel, Biel, Switzerland; ^5^ Department of Surgery, Kantonsspital Baden, 5404 Baden, Switzerland; ^6^ Department of Surgery, Klinikum rechts der Isar, Technische Universität München, Munich, Germany; ^7^ Department of Surgery, Tiefenau Hospital, Bern, Switzerland

**Keywords:** IL-23, colorectal cancer, prognosis, CD8

## Abstract

IL-23 is a heterodimeric cytokine involved in inflammatory diseases; its role in cancer progression is controversial. Here we analyse the expression of IL-23 subunits (p40 and p19) and IL-23R in colorectal cancer with regard to disease progression, clinical-pathological and molecular aspects. Immunohistochemistry for IL-23p19, IL-23p40, IL-23R and CD8 was performed on a multi-punch tissue microarray of 195 colorectal cancers (cohort 1), matched normal tissue, adenoma and lymph node metastases. Results were compared with clinical-pathological features and CD8+ T-cell counts, then validated on two patient cohorts (cohort 2: n=341, cohort 3: n=139). Cytoplasmic/membranous expression of IL-23 (p19 and p40 subunits) and IL-23R, respectively were over-expressed in carcinomas versus adenomas and normal tissues (p<0.0001) but were reduced in lymph node metastases (p<0.0001). Nuclear IL-23p19 expression was observed in 23.1% and was associated with early TNM stage (p=0.0186), absence of venous (p=0.0124) and lymphatic invasion (p=0.01493), favorable survival (p=0.014) and absence of distant metastasis (p=0.0146; specificity: 100%). This unexpected cellular localization was confirmed by cell fractionation. The beneficial effect of nuclear IL-23p19 was restricted to tumours with CD8+ high counts. Results were validated on Cohorts 2/3. This multicenter study underlines the possible CD8^+^ dependency and beneficial effect of nuclear IL-23p19 on overall patient survival.

## INTRODUCTION

IL-23 is a heterodimeric cytokine and member of the interleukin 12 superfamily [1] composed of an IL-23-specific subunit, IL-23p19 (IL-23A) and a subunit shared with IL-12 (IL-12p40). IL-23 is mainly secreted by activated dendritic cells and macrophages and plays an important role in mucosal immunology [2, 3]. It is widely known that IL-23 contributes to the progression of chronic inflammation by promoting the maturation and maintenance of T helper 17 cells (Th17) [4] and plays a pivotal role in the development of several autoimmune diseases like inflammatory bowel disease (IBD) [2], multiple sclerosis [5] and rheumatoid arthritis [6].

**Table 1:** Tissue microarray and clinicopathological data of included study cohorts

TMA characteristics		Cohort 1	Cohort 2	Cohort 3
	Origin:	Greece	Germany	Switzerland
	Total no. patients	N=213	N=341	N=139
	TMA Punch size	0.6mm	1.0mm	1.0mm
	No. punches	4/tumor	1/tumor	1/tumor
	Patients included from:	2002-2006	1993-2005	2002-2011
	Surgical specimen/Biopsy	Surgery	Surgery	Preoperative biopsy
Clinicopathological features				
Age (yrs)	Mean (min, max)	68.4 (35, 93)	66 (25- 91)	70.2 (30-93)
Tumour size (cm)	Mean (min, max)	4.6 (1, 12)	-	-
Gender	Male	99 (48.3)	193 (56.6)	87 (63.0)
	Female	106 (51.7)	148 (43.4)	51 (37.0)
Histological subtype	Non-mucinous	181 (88.3)	-	116 (86.6)
	Mucinous	24 (11.7)		18 (13.4)
Tumour grade	G1-2	130 (63.4)	225 (66.0)	81 (68.1)
	G3	75 (36.6)	116 (34.0)	38 (31.9)
Tumour location	Left	124 (60.5)	281 (82.4)	43 (31.4)
	Rectum	27 (13.2)	0 (0.0)	39 (28.5)
	Right	54 (26.3)	60 (17.6)	55 (40.2)
pT	pT1+pT2	51 (24.9)	79 (23.2)	37 (26.8)
	pT3+pT4	154 (75.1)	262 (76.8)	101 (73.2)
pN0	pN0	103 (50.2)	207 (60.9)	69 (50.0)
	pN1-2	102 (49.8)	133 (39.1)	69 (50.0)
pM	pM0	183 (89.3)	289 (84.8)	98 (73.1)*
	pM1	22 (10.7)	52 (15.3)	36 (26.9)*
TNM stage	Stage I	41 (21.8)	68 (20.0)	24 (17.9)
	Stage II	53 (28.2)	127 (37.4)	34 (25.4)
	Stage III	76 (40.4)	94 (27.7)	40 (29.9)
	Stage IV	22 (9.6)	51 (15.0)	36 (26.9)
	TNM staging ed.	6th	6th	7th
Tumour budding	Low-grade	115 (55.3)	-	71 (59.7)**
	High-grade	93 (45.7)		48 (40.3)
Venous invasion	Present	38 (18.1)	-	64 (53.3)
	Absent	172 (81.9)		56 (46.7)
Lymphatic invasion	Present	84 (40.6)	-	35 (29.9)
	Absent	123 (59.4)		82 (70.1)
Post-operative therapy	Untreated	76 (36.2)	209 (63.7)	108 (77.7)
	Treated	134 (63.8)	119 (36.3)	31 (22.3)
Pre-operative therapy	Untreated	210 (100%)	209 (100%)	117 (86.0)
	Treated	0 (0%)	0 (0%)	19 (14.0)
MMR status	Proficient	176 (91.7)	308 (90.3)	91 (85.1%)
	Deficient	16 (8.3)	33 (9.7)	16 (15%)
KRAS status	Wild-type	123 (65.1)	-	-
	Mutation	66 (34.9)		
BRAF status	Wild-type	182 (90.1)	-	-
	Mutation	20 (9.9)		
CIMP status	Negative/Low	94 (88.7)	-	-
	High	12 (11.3)		
Survival time	Median (95%CI)	60 (48-65)	120 (100-NE)	101 (62-NE)

Recent evidence suggests that IL-23-mediated responses are important in the promotion of tumour progression in a variety of tissue types. IL-23 signalling through its receptor can occur through activation of the JAK/STAT pathway, specifically through STAT3 leading to upregulation of proteins linked to proliferation and survival (bcl-x), angiogenesis, metastasis (VEGF, HIF1α, MMP2, MMP9) and immunosuppression [7]. Expression of IL-23 mRNA, and specifically of subunit p19, is upregulated in a variety of cancer types such as ovarian cancers, head and neck cancers, lung, breast and stomach cancers as well as melanoma in comparison to matched normal tissue controls [8]. *In vivo* studies also support the pro-tumour effect of IL-23 and its receptor as underlined by work on knock-out mice (IL-23R-/- or IL-23p19-/-) showing marked growth-restriction in transplanted tumours and reductions in angiogenesis [8-10]. A recent study shows that ablation of IL-23 in polyp-prone mice leads to a significant reduction in the number of colorectal polyps [11]. Also reported is a marked upregulation of IL-23 protein and mRNA in tumours from CPC-APC mice that develop cancers specifically in the colon. Grivennikov et al show diminished tumor multiplicity and growth with ablation of IL-23 or IL-23R in these mice [12].

On the other hand, anti-tumour and anti-metastatic properties of IL-23 have also been described. Lo and colleagues have shown that local production of IL-23 inhibits metastatic colorectal tumour growth *in vivo* and that this anti-tumour activity requires CD8+ T-cells, but not CD4+ T-cells or NK cells [13].

The aim of this study was to determine the expression of IL-23 (p19 and p40) and IL-23R in the progression of colorectal cancers through the adenoma-carcinoma-metastasis sequence, relationship to intratumoural CD8+ T-cells and impact on overall survival in patients with colorectal cancer.

## RESULTS

### Cohort 1

### Differences in expression of IL-23R, IL-12p40, IL-23 and nuclear IL-23p19 between tissue types

In a first step, differences in expression of IL-23R, IL-12p40, IL-23 and nuclear IL-23p19 between normal tissues, adenomas, primary colorectal cancers and lymph node metastases were investigated (Supplemental Figure 1). In all cases, there was a stepwise increase in expression from normal tissues to adenomas with the greatest expression in cancers (p<0.0001; all). Lymph nodes, on the other hand, showed a significantly lower expression in comparison to the primary tumour (p<0.0001; all).

### Association of IL-23R, IL-12p40, IL-23 and nuclear IL-23p19 with clinicopathological features and survival (Table 2)

**Table 2:** Association of nuclear IL-23p19 expression with clinicopathological data of Cohort 1

Feature		Total (n=213)	IL-23p19 N (%) (n=195)		P-value
			Negative (n=150; 76.9%)	Positive (n=45; 23.1%)	
Age (yrs)	Mean (min, max)	68.4 (35, 93)	68.2 (35, 93)	70.1 (41, 91)	0.2468
Tumour size (cm)	Mean (min, max)	4.6 (1, 12)	4.8 (1, 12)	4.1 (1.2, 8.0)	0.0333
Gender	Male	99 (48.3)	69 (47.6)	21 (47.7)	0.9869
	Female	106 (51.7)	76 (52.4)	23 (52.3)	
Histological subtype	Non-mucinous	181 (88.3)	128 (88.3)	41 (93.2)	0.5753
	Mucinous	24 (11.7)	17 (11.7)	3 (6.8)	
Tumour grade	G1-2	130 (63.4)	89 (61.4)	32 (72.7)	0.1695
	G3	75 (36.6)	56 (38.6)	12 (27.3)	
Tumour location	Left	124 (60.5)	87 (60.0)	22 (50.0)	0.4056
	Rectum	27 (13.2)	20 (13.8)	6 (13.6)	
	Right	54 (26.3)	38 (26.2)	16 (36.4)	
pT	pT1+pT2	51 (24.9)	31 (21.4)	17 (38.6)	0.0213
	pT3+pT4	154 (75.1)	114 (78.6)	27 (61.4)	
pN0	pN0	103 (50.2)	69 (47.6)	28 (63.6)	0.0621
	pN1-2	102 (49.8)	76 (52.4)	16 (36.4)	
pM	pM0	183 (89.3)	128 (87.7)	43 (100.0)	0.0146
	pM1	22 (10.7)	18 (12.3)	0 (0.0)	
TNM stage	Stage I	41 (21.8)	26 (17.3)	15 (33.3)	0.0186
	Stage II	53 (28.2)	40 (26.7)	13 (28.9)	
	Stage III	76 (40.4)	61 (40.7)	15 (33.3)	
	Stage IV	22 (9.6)	18 (12.4)	0 (0.0)	
Tumour budding	Low-grade	115 (55.3)	80 (54.8)	27 (60.0)	0.5385
	High-grade	93 (4.7)	66 (45.2)	18 (40.0)	
Venous invasion	Present	38 (18.1)	30 (20.3)	2 (4.4)	0.0124
	Absent	172 (81.9)	118 (79.7)	43 (95.6)	
Lymphatic invasion	Present	84 (40.6)	64 (43.8)	12 (27.3)	0.0493
	Absent	123 (59.4)	82 (56.2)	32 (72.7)	
Therapy	Untreated	76 (36.2)	49 (33.3)	22 (48.9)	0.0586
	Treated	134 (63.8)	98 (66.7)	23 (51.1)	
MMR status	Proficient	176 (91.7)	140 (95.2)	36 (80.0)	0.0032
	Deficient	16 (8.3)	7 (4.8)	9 (20.0)	
KRAS status	Wild-type	123 (65.1)	86 (65.2)	24 (60.0)	0.5522
	Mutation	66 (34.9)	46 (34.9)	16 (40.0)	
BRAF status	Wild-type	182 (90.1)	126 (90.0)	40 (88.9)	0.7835
	Mutation	20 (9.9)	14 (10.0)	5 (11.1)	
CIMP status	Negative/Low	94 (88.7)	77 (90.6)	17 (81.0)	0.249
	High	12 (11.3)	8 (9.4)	4 (19.1)	

Using Cohort 1, high expression of cytoplasmic and/or membranous staining was observed in 130/168 (77.4%) for IL-23R, 111/185 (60%) for IL-12p40 and 160/204 (78.4%) for IL-23 and was not associated with clinicopathological features or prognosis. In contrast, positive nuclear staining for IL-23p19 occurred in 45 tumours (23.1%) and was significantly associated with a smaller tumour diameter (p=0.0333), early pT classification (p=0.0213), earlier TNM stage (p=0.0186), absence of vascular invasion (p=0.0124), and absence of lymphatic invasion (p=0.0493). Most strikingly, of the 43 patients with IL-23p19 positivity, none had distant metastatic disease (p=0.0146). Sensitivity, specificity, positive predictive value (PPV) and negative predictive value (NPV) of IL-23p19 positivity for the absence of distant metastasis were 25.2%, 100%, 100%, and 12.3%.

### Nuclear IL-23p19 and survival

Positive expression of nuclear IL-23p19 had a significant and favorable effect on survival time, particularly at earlier time points. At 3-years, the overall survival rate was 87.5% (95%CI: 70-95) versus 64.7% (95%CI: 56-72) for negative patients (p=0.019). At 4-years, IL-23p19 positive patients had a 70% (95%CI: 28-91) overall survival rate in comparison to 55.6% (95%CI: 46-64) for negative patients (p=0.014). This effect on survival time was maintained in multivariable analysis with pT, pN and adjuvant therapy and indicated that the relative risk for patients with IL-23p19 positivity was 64% reduced in comparison to patients with IL-23p19 negative tumours (p=0.0321). Additionally, the effect was also not influenced by mismatch repair (MMR) status (p=0.034; HR (95%CI): 0.43 (0.2-0.94)). At 5-years, survival difference was no longer observed (Figure 2A-B).

**Figure 1:**
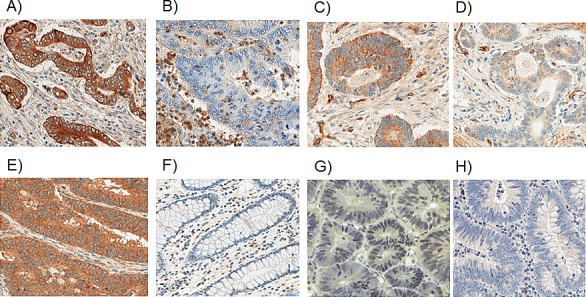
Representative photomicrographs of positive/high (A, C, E, G) and negative/low expression (B, D, F, H) of A-B: IL-23 poly; C-D: IL-23p40; E-F: IL-23R and G -H: IL-23p19. 100 × magnification.

**Figure 2:**
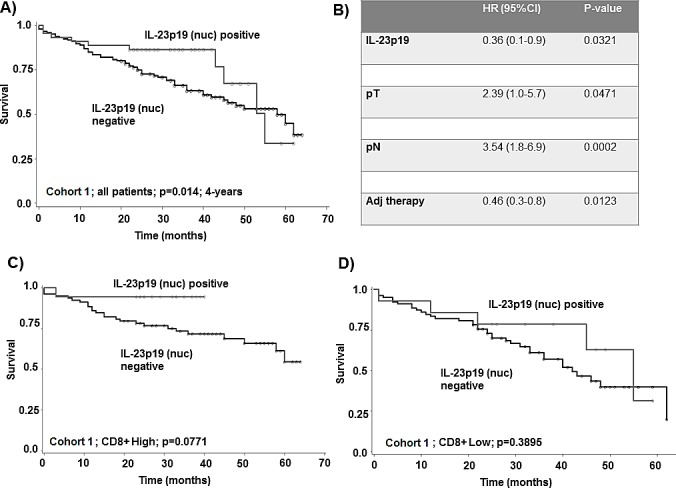
Cohort 3 A) Kaplan-Meier survival curve of test cohort showing survival time benefit of nuclear IL-23p19 positive patients particularly at early time points and B) a multivariable analysis of this cohort at 4-years follow-up. Stratification by CD8+ T-cell counts shows C) survival time benefit in patients with nuclear IL-23p19 positive cancers in CD8+ high setting with D) no benefit in CD8+ low setting.

### Association of IL-23p19, MMR, CIMP, KRAS and BRAF status

Molecular investigations of KRAS and BRAF revealed a 34.9% and 9.9% mutation rate, respectively. There was no association between IL-23p19 expression and KRAS or BRAF mutation. There was no association with high-level CIMP but a significantly larger number of nuclear IL-23p19 positive tumours were MMR-deficient (p=0.0032).

### Effect of nuclear IL-23p19 is dependent on the presence of CD8+ T-lymphocytes

There was no correlation between CD8+ T-lymphocytes and nuclear IL-23p19 expression. Since the effect of IL-23 on tumour growth restriction has previously been reported to depend on CD8+ T-lymphocytes [8], we stratified the cohort by total CD8+ counts using the median value. There were 96 patients in the CD8+ T-cell low group and 98 patients in the CD8+ T-cell high group. The associations between IL-23p19 nuclear staining and favorable prognostic features were exclusively maintained in the CD8+ T-cell high group (Table 3), namely with smaller tumour diameter (p=0.0001), earlier pT stage (p=0.022), lymph node negativity (p=0.0274), earlier TNM stage (p=0.0017) and absence of lymphatic invasion (p=0.033). Although not reaching statistical significance, all IL-23p19 nuclear positive cases (n=17) were free of distant metastasis. Overall survival time was also prolonged in patients with nuclear IL-23p19 positivity (100% survival) in the CD8+ T-cell high group (p=0.0771) (Figure 2 C-D) with no effect in the CD8+ T-cell low group (p=0.3895).

**Table 3:** Nuclear IL-23p19 in cohort 1 stratified by CD8+ low and high groups

Feature		CD8+ low (Freq N %)		P	CD8+ high (Freq N %)		P
		Negative (n=82)	Positive (n=14)		Negative (n=80)	Positive (n=18)	
Age (yrs)	Mean (range)	68.1 (36-91)	69.8 (41-83)	0.4168	68.9 (35-93)	68.2 (46-81)	0.7422
Tumour size (cm)	Mean (range)	4.8 (1-12)	5.0 (2.2-8.0)	0.6276	4.8 (1.2-9.0)	3.1 (1.2-6.0)	0.0001
Gender	Male	38 (48.1)	6 (42.9)	0.7172	38 (49.4)	7 (38.9)	0.4235
	Female	41 (51.9)	8 (57.1)		39 (50.7)	11 (61.1)	
Histological subtype	Non-mucinous	71 (91.0)	13 (92.9)	1.0	68 (87.2)	16 (88.9)	1.0
	Mucinous	7 (9.0)	1 (7.1)		10 (12.8)	2 (11.1)	
Tumour grade	G1-2	48 (61.5)	9 (64.3)	0.8454	49 (62.8)	15 (83.3)	0.0961
	G3	30 (38.5)	5 (35.7)		29 (37.2)	3 (16.7)	
Tumour location	Left	42 (53.9)	9 (64.3)	0.3591	51 (65.4)	7 (38.9)	0.1015
	Rectum	10 (12.8)	0 (0.0)		12 (15.4)	4 (22.2)	
	Right	26 (33.3)	5 (35.7)		15 (19.2)	7 (38.9)	
pT	pT1+pT2	17 (21.8)	4 (28.6)	0.7297	18 (23.1)	9 (50.0)	0.022
	pT3+pT4	61 (78.2)	10 (71.4)		60 (76.9)	9 (50.0)	
pN0	pN0	33 (42.3)	6 (42.9)	0.9694	43 (55.1)	15 (83.3)	0.0274
	pN1-2	45 (57.7)	8 (57.1)		35 (44.9)	3 (16.7)	
pM	pM0	67 (84.8)	14 (100.0)	0.2017	72 (92.3)	17 (100.0)	0.2371
	pM1	12 (15.2)	0 (0.0)		6 (7.7)	0 (0.0)	
TNM stage	Stage I	3 (3.9)	2 (14.3)	0.4088	2 (2.6)	5 (27.8)	0.0017
	Stage II	14 (18.0)	2 (14.3)		16 (20.2)	4 (22.2)	
	Stage III	48 (61.5)	7 (50.0)		43 (55.1)	(44.4)	
	Stage IV	13 (16.7)	3 (21.4)		17 (21.8)	1 (5.6)	
Tumour budding	Low-grade	41 (51.3)	9 (64.3)	0.3672	46 (59.0)	11 (61.1)	1.0
	High-grade	39 (48.8)	5 (35.7)		32 (41.0)	7 (39.0)	
Venous invasion	Present	20 (24.7)	1 (7.1)	0.144	11 (13.9)	0 (0.0)	0.0927
	Absent	61 (75.3)	13 (92.9)		68 (86.1)	18 (100.0)	
Lymphatic invasion	Present	38 (48.1)	6 (42.9)	0.7172	29 (37.2)	2 (11.1)	0.033
	Absent	41 (51.9)	8 (57.1)		49 (62.8)	16 (88.9)	
Therapy	Untreated	18 (22.2)	6 (42.9)	0.1787	34 (43.6)	12 (66.7)	0.0773
	Treated	63 (77.8)	8 (57.1)		44 (56.4)	6 (33.3)	
MMR status	Proficient	76 (95.0)	9 (64.3)	0.0003	75 (94.9)	15 (83.3)	0.1164
	Deficient	4 (5.0)	5 (53.7)		4 (5.1)	3 (16.7)	
KRAS status	Wild-type	40 (56.3)	8 (61.5)	0.7708	52 (71.2)	9 (64.3)	0.7507
	Mutation	31 (43.7)	5 (38.5)		21 (28.8)	5 (35.7)	
BRAF status	Wild-type	70 (92.1)	13 (92.9)	1.0	67 (88.2)	15 (83.3)	0.6942
	Mutation	6 (7.9)	1 (7.1)		9 (11.8)	3 (16.7)	
CIMP status	Negative/Low	39 (88.6)	9 (90.0)	0.9014	38 (82.7)	8 (72.7)	0.1006
	High	5 (11.4)	1 (10.0)		3 (7.3)	3 (27.3)	

### Validation study

### Cohort 2: Frequency and prognostic impact of nuclear IL-23p19 expression

Nuclear IL-23p19 positivity was identified in 46/341 (13.5%) of cases and was significantly associated with a longer follow-up time (p=0.0116). A tendency toward an earlier TNM stage (p=0.0977) was observed. Most importantly, of the 46 tumours with IL-23p19 positivity, only 3 had metastatic disease in comparison to 49 of the 295 negative patients (p=0.0529). Sensitivity, specificity, PPV and NPV of IL-23p19 for distant metastasis were 14.9%, 94.2%, 93.4%, and 16.6%, respectively. Survival time in patients with nuclear IL-23p19 positivity was considerably longer in comparison to IL-23p19 negative patients, but did not reach significance. However, a subgroup analysis of patients by TNM stage revealed a significant benefit in patients with stage III disease (p=0.0363) (Supplemental Figure 2). This association was also maintained in multivariable analysis with adjuvant therapy (p=0.0413; HR (95%CI): 0.29 (0.1-0.9)). Moreover, of these patients, 10 were MMR-deficient. There was no association between IL-23p19 and MMR status. Adjusting the multivariable analysis for MMR status, again IL-23p19 positivity had a significant beneficial effect on overall survival (p=0.0446; HR (95%CI: 0.35 (0.12-0.98)).

### Cohort 3: Impact on survival stratified by CD8+ T-lymphocytes

**Figure 3:**
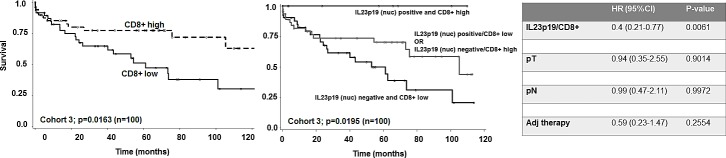
A) Kaplan-Meier survival curve of low and high CD8+ T-cell counts in patients not receiving a preoperative therapy (n=100). B) Kaplan-Meier curve showing combined nuclear IL-23p19 and CD8+ T-cell counts and effect on overall survival. Nuclear IL-23p19 positive/CD8+ high patients have a 100% survival. Effect of IL-23p19 negative/CD8+ high or IL23-p19 positive/CD8+ low has a more unfavorable intermediate effect while IL-23p19 negative/CD8+ low patients have the most limited benefit. C) Multivariable analysis showing the independent prognostic effect of the combined IL-23p19 positive/CD8+ effects on overall survival.

Nuclear IL-23p19 positivity was identified in 22/139 (15.8%) of cases. There was no association of expression with clinicopathological features. Evaluating only patients who did not receive any preoperative therapy (n=117), survival time analysis showed a marked favorable survival time in patients expressing nuclear IL-23p19 but this result did not reach statistical significance (p=0.1536). Cases were then stratified into low and high intraepithelial CD8+ T-cell counts (median value as threshold: 3.4 cells). Evaluating only patients with both CD8+ T-cell counts and IL-23p19 counts (n=100), a beneficial effect of IL-23p19 positivity on overall survival appears limited to the CD8+ high group (Supplemental Figure 2). This effect is underlined when evaluating the combination of IL-23p19 and CD8+ groups (p=0.0195). A clear additive effect of IL-23p19 to CD8+ T-cell counts can be observed by comparing Figures 3A and 3B. 5-year overall survival for patients with both IL-23p19 nuclear expression and CD8+ high counts was 100% in comparison to those with IL-23p19 negative and CD8+ low counts (39.2% (95%CI: 21-57)) and 70% (95%CI: 53-82) for patients with either IL-23p19 negative/CD8+ high or IL-23p19 positive/CD8+ low counts (Figure 3B). This effect could even be maintained in multivariable analysis with pT, pN and adjuvant therapy (Figure 3C). Additionally, information on MMR status was available for 93 of these patients and 15/93 (16.1%) were identified as MMR-deficient. Of these 15 patients, 3 were positive for nuclear IL-23p19 and 12 negative (p=0.7177). Not only did multivariable analysis of the combined expression IL-23p19/CD8+ with MMR status maintain its statistical significance (HR (95%CI: 0.39 (0.2-0.73); p=0.0035)), but subgroup analysis also showed similar survival times after removal of –deficient cases (p=0.0155).

### Cell fractionation and confirmation of nuclear expression

**Figure 4:**
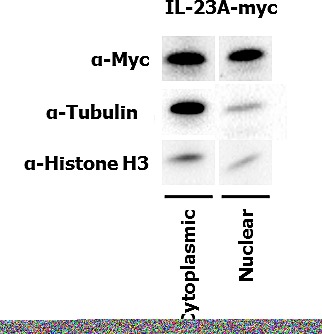
Immunoblot showing both nuclear and cytoplasmic fractions for IL-23p19 (IL-23A) and appropriate controls.

In order to confirm the localization of expression of IL-23p19, a myc-tagged IL-23p19 expression plasmid was used. Figure 4 shows the localization of the IL23p19 protein in both cytoplasmic and nuclear fractions with both localizations producing strong bands upon immunoblotting.

## DISCUSSION

In this study we could demonstrate that a) IL-23 signalling may be involved in the early, rather than late, progression of colorectal cancer, b) nuclear expression of IL-23p19 is a marker of early disease and c) the beneficial prognostic impact of nuclear IL-23p19 may be restricted to patients with high anti-tumour T-cell responses. This was accomplished by first investigating IL-23p19, IL-23p40 and IL-23R in normal colonic tissue, matched adenomas, carcinomas and lymph node metastases from a well-characterized cohort of colorectal cancer patients. Then a total of 675 patients from three independent institutions were analysed and correlated with overall survival and CD8+ T-cell counts.

Our findings underline an increased expression of IL-23p19, IL-23p40 and IL-23R in the normal-adenoma-carcinoma sequence followed by a marked reduction in expression in lymph node metastases. This result supports the notion that IL-23 and its receptor may be required for early colorectal tumourigenesis rather than for metastatic dissemination. This result is further supported by the association of nuclear IL-23p19 with early TNM stage, favourable survival time and the notable absence of distant metastatic disease. Our results are in line with Trinchieri and colleagues who showed a significant upregulation of IL-23 mRNA (p19 and p40) in various cancer tissues in comparison to their adjacent normal tissue counterpart [20]. Langowski et al found similar results highlighting an increased IL-23p19 gene expression in colon cancers in comparison to normal tissues [8]. Recently, Grivennikov and co-workers found an upregulation of IL-23 in mouse colon tumours in comparison to matched non-tumour colons [12]. Others have shown increased IL-23 levels in the serum of colorectal cancer patients in comparison to healthy controls [21]. Interesting is also the apparent change of expression of IL-23p19 from a more cytoplasmic to nuclear localisation with progression from adenomas to carcinoma. While such a change in protein expression by cellular localisation is not unusual (e.g. β-catenin), it may be congruent here with a change in the inflammatory response. Our study using matched normal-adenoma-carcinoma and lymph node metastases underlines the possible role of IL-23/IL-23R in the early, rather than late progression of colorectal cancers.

Although our results point to a beneficial impact of IL-23p19 on patient outcome, controversial and opposing roles of IL-23 have been previously described. On the one hand, Langowski and colleagues show that IL-23-receptor-deficient mice show an impressive growth-restriction in transplanted tumours. Moreover, the expression of MMP9, a marker of tumour-angiogenesis is significantly decreased in IL-23p19-/- mice [8]. On the other hand, several studies point toward a role for IL-23 in enhanced anti-tumour immunity. Lo and colleagues show that local IL-23 secretion reduces tumourigenicity and metastasis, promotes growth retardation and prolongs survival in a mouse model [13]. Mice over-expressing IL-23 were found to secrete large amounts of IFNγ, suggesting the involvement of T-cells, leading to systemic anti-tumour immunity [22]. This was further supported by work by Shan et al. showing that IL-23 secreted by tumour cells could enhance CTL activity and the production of Th1 cytokines such as IFNγ, IL-12 and TNF-α [23].

In our study, we show that the beneficial prognostic effect of nuclear IL-23p19 on overall survival may be restricted to tumours with high CD8+ T-cell counts. Possibly, IL-23 could induce an enhancement of CD8 activity, shifting the balance within the tumour microenvironment towards an anti-tumourous milieu [23, 24]. This finding is underlined by the more frequent IL-23p19 positivity in tumours with MMR-deficiency, a subgroup of colorectal cancers known for their abundant CD8+ T-cell counts and favourable survival in affected patients. Our finding is in agreement with previous *in vivo* experiments investigating effects of injection of IL-23p19 over-expressed colon cancer cell lines into either immune-competent or -deficient mice. In BALB/c mice, IL-23p19 over-expression in cell lines and injection led to a significantly lower tumour volume in comparison to control [22]. When these mice were treated with anti-CD8 antibody, this effect was compromised [13]. In SCID (deficient in B and T cells) or SCID/beige mice (deficient in B, T and NK cells), no difference in effects was observed with or without IL-23p19 over-expression. These results suggest that the anti-tumour properties of IL-23p19 may be found in tumours with abundant CD8+ T-cell counts. In our study, the beneficial effects of nuclear IL-23p19 on overall patient survival are also generally restricted to patients with high CD8+ T-cell numbers. In fact, although CD8+ T-cell counts alone have been shown to have an excellent prognostic impact [18], an additive effect leading to improved stratification of patients with nuclear IL-23p19 positivity among those with high CD8+ T-cell counts is highlighted in this study.

It is known that several molecular features in colorectal cancers may have an influence of the immune response. In the case of MMR-deficiency, colorectal cancers often show an inherent abundance of CD8+ T-cells. These tumors which are microsatellite instable are often BRAF mutated and show a high-level CIMP, but rarely KRAS mutations [25]. Because of its role in immunity, these molecular features were evaluated in the context of IL-23 to ensure no confounding of our results. A recent work by Petanidis and colleagues showed that inhibition of KRAS using manumycin A could cause a significant decrease in IL-23 levels establishing a correlation between these two molecules [26]. We found no association between IL-23p19, and these molecular changes. This is similar to Grivennikov and colleagues who report an upregulation of IL-23 in early cancers but no further increase in expression in more advanced mouse colorectal cancers caused by Apc loss and forced KRAS or BRAF activation [12].

Nuclear expression of IL-23p19 using a monoclonal antibody was found in 13-23% of all patients. This unexpected nuclear localisation was nonetheless confirmed after cell fractionation that showed both cytoplasmic and nuclear expression. The result is consistent with the findings using both the polyclonal (IL-23) and monoclonal (IL-23p19) antibodies targeting this protein by immunohistochemistry. The function, however, of IL-23p19 in the nucleus remains to be elucidated. Since cytokines secreted outside of their original compartment can no longer be observed by immunohistochemistry, the observation of nuclear localisation does not preclude stimulation of other cell types after secretion. In this study, we tested the clinical relevance of IL-23p19 nuclear expression in 675 patients from three different countries (Greece, Germany and Switzerland). Interestingly, consistent survival results were found, suggesting that the biological behaviour of the tumours may, in this case, be more relevant to patient outcome than standard surgery. These results could furthermore be found using multiple- or single punch tissue microarrays and also using material from resection specimens and also biopsies. Some limitation may nonetheless include the limited availability of some clinical/pathological features in the validation cohorts including.

In conclusion, IL-23 and IL-23R may play a role in early colorectal cancer progression. However, among patients with cancer, nuclear expression of IL-23p19 is an advantageous prognostic factor with an apparent ‘protective’ function that may be CD8+ dependent. Additional studies are required to confirm these findings in colorectal cancer patients and to further elucidate the role of IL-23p19 in tumour cells and link to anti-tumour immunity.

## MATERIALS AND METHODS

### Patients

### Clinicopathological data of all patient cohorts can be found in Table 1

Cohort 1 (Test cohort)

A retrospective study cohort of 220 non-consecutive patients with primary resected colorectal cancers treated at the Fourth Department of Surgery, University of Athens Medical School in Athens, Greece, between 2002 and 2006 was entered into this study. Clinicopathological data were reviewed from patient charts and included age at diagnosis, gender, tumour location, the histological subtype of the tumour, tumour grade, TNM stage (UICC 6^th^ ed.), and the presence of lymphatic and vascular invasion. Additionally, tumour budding at the invasion front was assessed using a 10 in 10 method and classified into high-grade and low-grade [14]. None of the patients received pre-operative therapy. No patient had IBD-associated colorectal cancers. Information on post-operative therapy was available for 210 patients. Median survival time was 60 months (95%CI: 48-65). The clinical endpoint of interest was distant metastasis and overall survival. Permission has been granted by the local ethics committee of the University of Athens.

### Cohort 2 (Validation cohort on resection specimens)

In order to validate important results from the test cohort, a second cohort consisting of 341 primary resected colon cancer (no rectal cancer) patients treated at the Department of Surgery at the Technical University Munich hospital, Munich, Germany, between 1993 and 2005 was used. Clinical and pathological features for this cohort included age at diagnosis, gender, tumour location, TNM stage (UICC 6^th^ ed), R classification, and tumour grade. Information on post-operative therapy was available in 328 cases. Seven patients had an IBD-associated colorectal cancer. Median survival time for the cohort was 120 (95%CI: 100-NE) months. The clinical endpoint of interest was distant metastasis and overall survival. The ethics committee of the Klinikum rechts der Isar approved the study (no. 1926/7). All samples were obtained after prior informed written consent.

### Cohort 3 (Validation cohort on preoperative biopsies)

Next, in order to validate findings from cohorts 1 and 2 on a third independent patient sample this time using preoperative colorectal cancer biopsies, we identified 139 patients with both biopsy and matched colorectal cancer resection treated from 2002-2011 at the Bern University Hospital (Bern, Switzerland). All Haematoxylin and Eosin (HE) slides from surgical resections were re-reviewed. Information on age at diagnosis, gender, tumour location, tumour grade, and TNM (UICC 7^th^ ed.) was recorded. Nineteen patients received a pre-operative therapy. No patients had an IBD-associated colorectal cancer. Information on post-operative therapy and survival time was available. Median survival time was 101 (95%CI: 62-NE) months. Clinical endpoint of interest was overall survival. Permission has been granted by the local ethics committee (Registration: 07-10-13).

### Assay Methods

### Tissue microarray

Tissue microarrays were used for evaluation of protein expression. For Cohort 1, a multiple-punch tissue microarray of 0.6mm diameter cores was investigated. On average 4 tumour punches were obtained per case including 2 from the tumour center and 2 from the invasion front [15]. Single tissue punches of 54 adenomas, 151 normal tissues and 88 tumour infiltrated lymph nodes were also included. The complete array contained 1283 tissue spots. For the Cohort 2, a single-punch tissue microarray of 1.0mm diameter cores per colon cancer was evaluated [16]. For Cohort 3, a single-punch tissue microarray of 1.0mm in diameter was constructed based on preoperative biopsy material [17].

### Immunohistochemistry

A multi-tissue test microarray containing 280 tissue spots from matched neoplastic and non-neoplastic tissues from different tissue types and organs was initially used for antibody testing and validation. This tissue microarray was cut at 4μm and underwent immunohistochemistry using an automated immunostainer (Leica, Bond III) for the following antibodies: IL-23R (Abcam, goat polyclonal, citrate 30', 100°, 1:100), IL-12p40 (aka IL-12B, Abcam, rabbit polyclonal, Tris 30', 95°, 1:2500), IL-23 (aka IL-23p19, Abcam, rabbit polyclonal, no pretreatment, 1:200) and IL-23p19 (Abcam, mouse monoclonal, clone HLT 2736, enzyme pretreatment, 1:25). Next, the tissue microarray for the Cohort 1 was similarly cut and immunostaining performed for all antibodies. Additional staining for CD8+ (Abcam, Tris 20' 95°, 1:100) was performed on the Cohort 1 and Cohort 3 [17].

### Evaluation of immunohistochemistry

For evaluation of immunohistochemistry, only epithelial cells were scored. Membranous and/or cytoplasmic immunoreactivity was noted for IL-23R, IL-12p40, and IL-23. In addition, nuclear staining was observed for IL-23 using the monoclonal antibody (IL-23p19). For the normal tissue, adenoma and lymph node, the percentage of positive cells was denoted. The percentage of positive IL-23 cells per punch was investigated and then the average expression across the multiple cores was assigned per patient. Then, using receiver operating characteristic (ROC) curve analysis, each patient was assigned a negative and positive scores based on the identified threshold value. For IL-23R, IL-12p40 and IL-23, this threshold was 95% (low expression <95%, full expression ≥95% tumour cell staining). Nuclear staining of IL-23p19 was classified as negative (0%) or positive (any staining). Representative immunostains are shown in Figure 1. Although this study focused on cancer cell expression of these markers, stromal cell staining was observed for IL-12p40, IL-23R and IL-23 (polyclonal antibody). Only very infrequent expression of nuclear IL-23p19 was observed in dendritic cells in colorectal cancers and was considered too rare to be evaluated separately. CD8+ staining was evaluated on whole tissue sections from biopsies in cohort 3 in 5 HPFs as found in [17] and the average across the 5 fields used for analysis. For Cohort 1, intraepithelial and stromal counts of CD8+ were made per punch, pooled and then averaged across each tumour [18]. For Cohort 3, only intraepithelial counts were made, since these were found on the same patient cohort to have the greatest prognostic significance [17].

### Molecular analysis

Since the molecular background of colorectal cancers may influence the immune response, analysis of KRAS and BRAF mutations, as well as CpG Island Methylator Phenotype (CIMP) were carried out. Detailed protocols and primer sequences can be found in [19]. Briefly, mutation analysis by pyrosequencing for *BRAF* (exon 15, V600E mutations) and *KRAS* (exon 2, codon 12 and 13) was performed. For CpG methylation analysis, bisulfite conversion of all DNA samples was undertaken using the EpiTect Bisulfite Conversion Kit (Qiagen) according to manufacturer's instructions. CpG analysis for *SOCS1*, *NEUROG1, MLH1, CRABP1A, CDKN2A*, and *RUNX3* were also carried out. Following PCR, fragment analysis was carried out using the QIAxcel system (Qiagen). Bisulfite controls were included into the program for each assay manually to ensure complete conversion of DNA. For each assay, the ratio of C:T indicating the percentage of methylated to non-methylated C residues at each CpG site was noted. Then an average percentage of methylation across each case was determined. For each gene, the 75^th^-percentile was used as a cutoff to determine “hypermethylation” status. CIMP-high was defined by at least 3/6 hypermethylated genes, CIMP-low as at most 2/6 hypermethylated genes and CIMP-negative as 0/6 hypermethylated genes.

### Expression plasmids and transient transfection

A myc-tagged IL-23p19 (IL-23A) pCMV6 expression plasmid was purchased from Origene (Labforce, Nunningen, Switzerland) and 4 μG of this plasmid were transfected into 293T HEK cells using calcium phosphate technique.

### Cellular fractionation and immunoblotting

Cytoplasmic and nuclear fractionation was achieved by first lysing the cells in buffer A (HEPES 10 mM (pH 8.0), KCl 10 mM, MgCl2 1.5 mM, DTT 1mM), centrifugation and recovering the supernatant. The cell pellet was re-suspended in buffer B (HEPES 20 mM (pH 8.0), MgCl2 1.5 mM, KCl 400 mM, NaCl 40 mM, EDTA 0.2 mM, DTT 1 mM, glycerol 25%) to lyse the nuclear envelope and nuclear proteins were extracted. Protein content was determined by a Bradford Assay (#500-0006; Bio-Rad) with BSA as a standard. 5μg of cytoplasmic and nuclear protein was analysed by electrophoresis on a mini protean TGX Precast gel (Bio-Rad). Primary antibodies used were anti-Myc-tag (AB13836; Abcam), anti-α-Tubulin (#3873; Cell Signaling) and anti-Histone H3 (06-599; Millipore). Secondary antibodies used were anti-rabbit and anti-mouse IgG HRP-linked (NA93IV; GE Healthcare). Chemiluminescence signal was detected using an ECL-kit (170-5060; Bio-Rad).

### Statistical Analysis

Differences in expression between tissue types were investigated using the Friedman test for matched samples and Kruskal-Wallis test. Differences in expression and association with categorical clinicopathological features were analysed with the Chi-Square or Fisher's Exact test, where appropriate. For patient age and tumour diameter, the Wilcoxon Rank Sum Test was used. Kaplan-Meier curves were plotted and survival time differences assessed using the log-rank test. Multivariable analysis was performed by including possible confounders and post-operative therapy in addition to the protein marker of interest into a Cox regression analysis. Hazard ratios (HR) and 95% confidence intervals (CI) were used to determine the effect of each variable on overall survival. The sensitivity, specificity, positive and negative predictive values (PPV, NPV) of the expression for distant metastasis were identified. Correlations with CD8 were made using the Spearman Rank correlation coefficients. All p-values were two-sided and considered significant when p<0.05. Analyses were performed using SAS (V9.2, The SAS Institute, Cary, NC).

## SUPPLEMENTARY FIGURES


