# Supplementary material for: Investigation of IL-23 (p19, p40) and IL-23R identifies nuclear expression of IL-23 p19 as a favorable prognostic factor in colorectal cancer: a retrospective multicenter study of 675 patients
Source: Oncotarget. 2014 Jun 6;5(13):4671–82. doi: 10.18632/oncotarget.2069 (PMC4148090; doi:10.18632/oncotarget.2069)
Supplement: Supplementary file 1 [file oncotarget-05-4671-s001.pdf]

Investigation of IL-23 (p19, p40) and IL-23R identifies nuclear expression of IL-23 p19 as a favorable prognostic factor in colorectal cancer: a retrospective multicenter study of 675 patients

Supplementary Material

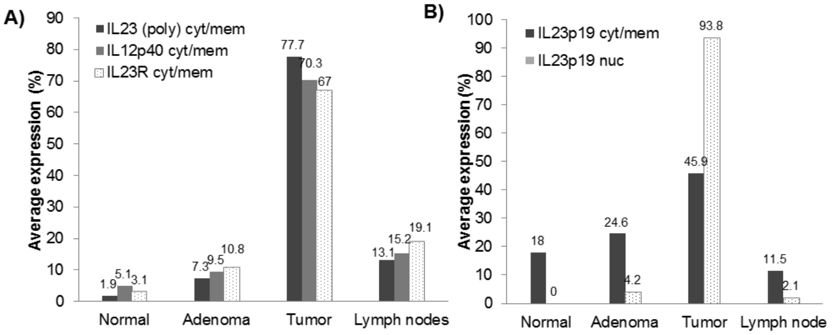

Supplementary fig1:

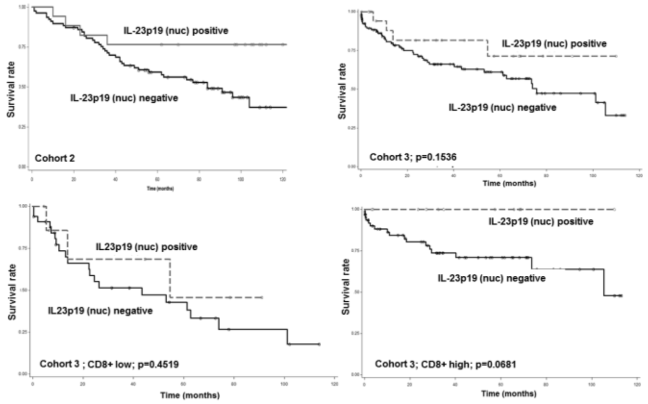

Supplementary fig2:
